# Hyperpolarization‐Activated Cyclic Nucleotide‐Gated Channels and Ventricular Arrhythmias in Heart Failure: A Novel Target for Therapy?

**DOI:** 10.1161/JAHA.113.000287

**Published:** 2013-06-21

**Authors:** Priyanthi Dias, Cesare M. Terracciano

**Affiliations:** 1 Imperial College London, National Heart and Lung Institute, Imperial Centre for Translational and Experimental Medicine, Hammersmith Hospital, London, UK (P.D., C.M.T.)

**Keywords:** editorials, ivabradine, heart failure, HCN channels, funny current, arrhythmias

## Introduction

Arrhythmias are a common and often fatal complication of heart failure accounting for 50% to 70% of cardiac deaths due to tachyarrhythmic mechanisms.^1^ In the failing heart, disturbances in cardiac rhythm are the likely result of cardiac remodeling consisting of structural and/or electrophysiological abnormalities. The structural heterogeneities provide a substrate for triggered activity from which ventricular arrhythmias can be initiated. Pathological alterations in ion channel properties and gap junction intercellular communication lead to changes in the action potential profile and cellular excitability, serving as a trigger for arrhythmias. Although extensive pharmacological therapies have been developed for the treatment of arrhythmias, efficacy of the drugs remains limited due to the complex remodeling processes underlying heart failure.

Among the ionic mechanisms that contribute to ventricular arrhythmias, the role of the pacemaker or funny current (*I*_f_) and the hyperpolarization‐activated cyclic nucleotide gated (HCN) channels is emerging. The subject has been addressed by Kuwabara et al^2^ in this issue of *Journal of the American Heart Association*.

*I*_f_ is a mixed Na^+^‐K^+^ inward current slowly activated by membrane hyperpolarization, associated with generating automaticity in the sinoatrial node.^3^ In addition to voltage, activation of *I*_f_ is also modulated by catecholamines and intracellular cyclic nucleotides such as cyclic AMP (cAMP). For instance, increasing cAMP levels via β‐adrenergic stimulation causes a rightward shift in *I*_f_ activation meaning that the current is activated at more positive voltages without affecting the maximum current amplitude. This mechanism participates in the relationship between β‐adrenergic stimulation and regulation of heart rate.^3^

Over the last 20 years, much attention has focused on *I*_f_ in nonpacemaker cells and its potential role in triggering ventricular arrhythmias in both patients and various experimental animal models.

During fetal and neonatal development *I*_f_ is abundantly present in ventricular myocytes and progressively declines during maturation.^4^ Thus, in the adult heart, *I*_f_ can be detected only in a minority of ventricular myocytes, and the current is significantly reduced compared with neonatal myocytes. Under certain pathological conditions *I*_f_ is able to recover its “fetal” phenotype with an increase in the numbers of ventricular myocytes in which the current can be recorded and also with greater current amplitude. This has been demonstrated extensively during cardiac hypertrophy in both humans and several animal models of heart failure, such as pressure overload and postmyocardial infarction.^5–6^ Interestingly, there is a strong degree of correlation between the severity of hypertrophy and *I*_f_ density in ventricular myocytes; the expression of the HCN channels responsible for *I*_f_ is most pronounced in regions with the greatest overload,^7^ raising the possibility that this mechanism is mechano‐sensitive. The nature of the initial injury also seems a determining factor in this phenomenon. Ischemic cardiomyopathy for example leads to a greater *I*_f_ current density in ventricular myocytes compared with dilated cadiomyopathy.^8^ Moreover, the link between β‐adrenergic stimulation and *I*_f_ may have a role in these differences and could provide a potential arrhythmogenic mechanism in a setting of enhanced sympathetic drive such as heart failure, with an increased contribution of the current during diastole in ventricular myocytes.

The molecular determinants of *I*_f_ in both healthy and diseased hearts are the HCN channels responsible for generating the current. There are 4 known isoforms identified in mammals (HCN1 to 4) of which HCN1, HCN2, and HCN4 are differentially expressed throughout the myocardium but with the highest levels in the sinoatrial node. The adult ventricle has low levels of HCN2 and HCN4 which subsequently increase at both the mRNA and protein levels as a result of heart failure.^6–7^ The specific overexpression of HCN1 has also been reported recently in heart failure.^9^ It is possible that increases in the different HCN isoforms that are normally present at low levels could potentially interact and give rise to heterotetramers with unique biophysical and regulatory properties compared with homomers. Posttranscriptional mechanisms may also contribute to HCN expression through microRNA.^10^ Collectively this increase in HCN subunit expression is likely to be the underlying cause for the gain of function in *I*_f_ recorded from diseased ventricle.

The majority of studies have focused on the modifications in functional and molecular properties of *I*_f_ and HCN channel expression during cardiac remodeling, yet the precise involvement of *I*_f_ in arrhythmias remains to be fully determined. The paper by Kuwabara et al^2^ provides insight into the role of *I*_f_ in the initiation of triggered activity in a bradycardic model of heart failure. By using genetically modified mice that express a dominant negative form of the transcriptional repressor neuron‐restrictive silencing factor (dnNRSF‐Tg), the authors generated a model of heart failure^11^: NRSF, a regulator of the fetal cardiac gene program, activates the reexpression of HCN2 and HCN4 to induce dilated cardiomyopathy. Kuwabara et al tested the hypothesis that expression of HCN channels predisposes ventricular myocytes to enhanced automaticity. The major findings of the study are the increase in *I*_f_ density and the high incidence of ventricular tachycardia, premature ventricular contractions, and sudden cardiac death in the dnNRSF‐Tg mice. β‐adrenergic stimulation increased the susceptibility of myocytes to early after‐depolarizations and spontaneous action potentials through increased cAMP production. These findings were confirmed in myocytes from mice with cardiac specific overexpression of HCN2, demonstrating a link between HCN and arrhythmias. These results are also in agreement with another study^9^ showing that HCN activity increases the proarrythmogenic potential in the failing myocytes through prolongation of the repolarization phase in ventricular action potentials.

To confirm the contribution of HCN overexpression in the development of arrhythmias, Kuwabara et al^2^ used ivabradine, a selective heart‐rate–reducing agent, to inhibit channel activity. Ivabradine blocks HCN activity by entering the pore of the channels when they are in open state thereby reducing the slope of diastolic depolarization.^12^ A major clinical trial to demonstrate the beneficial effects of ivabradine is the BEATUTIFUL (morBidity–mortality EvAlUaTion of the *I*_f_ inhibitor ivabradine in patients with coronary disease and left ventricULar dysfunction) study treating 10 917 patients with stable coronary artery disease and left ejection fraction <40%. Although ivabradine treatment did not affect the primary composite outcome, it reduced the need for hospitalization due to myocardial infarction and coronary revascularization in patients with baseline heart rates >70 bpm.^13^ This work was followed by the SH*I*_f_T (Systolic Heart failure treatment with the *I*_f_ inhibitor ivabradine Trial) trial (6558 patients), which confirmed that treatment with ivabradine significantly reduced hospital admission for worsening heart failure by 18% and cardiovascular deaths due to heart failure.^14^ In both trials the favorable effects of ivabradine were greatest in those patients with a higher heart rate. The most striking finding of the SH*I*_f_T trial, obtained from the echocardiographic substudy, was the reversal of cardiac remodeling in the left ventricle with pronounced increase in left ventricular ejection fraction and reduced left ventricular systolic volume.^15^ Effects of ivabradine in preventing arrhythmias were also investigated in a small group of patients (21) with inappropriate sinus tachycardia. Ivabradine reduced 70% of symptoms reported at baseline and in 50% of patients all symptoms were eliminated highlighting its potential use in the treatment of arrhythmias.^16^ Beneficial effects of chronic treatment with ivabradine have also been shown in rodent models of heart failure with improvements in whole heart systolic and diastolic function, reduction in interstitial and perivascular fibrosis, increase in angiogenesis and myocardial perfusion, improved calcium handling, decreased mortality and ventricular excitability, and reversal of myocardial structural remodeling.^17–19^

The mechanisms of ivabradine, however, remain controversial. Most clinical and experimental evidence points to a primary role of heart rate reduction. Ivabradine‐driven cardiac remodeling has been extensively studied at doses that lower heart rate and the reduction in heart rate is thought to be the likely cause of remodeling by optimizing energy consumption while simultaneously reducing cardiac work load.^20^ However, there are studies showing that some of the effects may be mediated by different mechanisms. We have recently shown that the reduction in myocardial fibrosis induced by ivabradine is not due to heart rate reduction, as the β‐adrenergic blocker metoprolol did not reduce fibrosis in a rodent model of heart failure, despite a similar reduction in heart rate.^19^ Kuwabara et al^2^ also studied the remodeling effects on arrhythmias independent of heart rate reduction by using a nonbradycardic dose of ivabradine. With this strategy they clearly demonstrated the reduction in *I*_f_ amplitude and occurrence of spontaneous action potentials in the presence of isoproterenol and improved survival rate in the dnNRSF‐Tg model. Other effects of ivabradine independent of heart rate include the attenuation of the renin–angiotensin aldosterone system (RAAS) through a reduction in angiotensin‐converting enzyme and angiotenisn II type I receptor (AT‐1) transcript expression.^17^ All these studies support the notion that ivabradine can have direct effects on ventricular structure and electrophysiology independent of the sinoatrial node.

The paper by Kuwabara et al^2^ contributes to the significant progress that has been made in establishing the role of *I*_f_ and HCN channels as markers of pathological remodeling and identifying the cardioprotective effects of ivabradine in the failing heart. However, there are key questions that remain unanswered: (1) Is the overexpression of HCN channels and augmented *I*_f_ in the ventricle an adaptive response with specific functional consequences or is it simply part of a global remodeling process? (2) What is the role of the different HCN isoforms and how do the isoforms contribute to the clinical and pathological scenario? (3) What are the effects of ivabradine on the fibrotic pathways, in particular the RAAS system and the local regulation of ECM? (4) Are the effects of ivabradine on arrhythmias secondary to the reversal of remodeling rather than a direct effect on the HCN channels? (5) What are the reasons for the variability in the effects of ivabradine in different HF models and at different stages of disease?

Pharmacological inhibition of the HCN channels remains a potentially useful strategy in heart failure as there are already significant encouraging results from large clinical trials and accumulating positive laboratory studies. However, more specific clinical trials should address the efficacy on treating arrhythmias in heart failure and it is also essential that the specific mechanisms involved are investigated in the laboratory in order to understand the pathways involved and refine this novel and promising approach.

## References

[1] Goldberger JJ Buxton AE Cain M Costantini O Exner DV Knight BP Lloyd‐Jones D Kadish AH Lee B Moss A Myerburg R Olgin J Passman R Rosenbaum D Stevenson W Zareba W Zipes DP Risk stratification for arrhythmic sudden cardiac death: identifying the roadblocks. Circulation. 2011; 123:2423-2430 21632516 10.1161/CIRCULATIONAHA.110.959734

[2] Kuwabara Y Kuwahara K Takano M Kinoshita H Arai Y Yasuno S Nakagawa Y Igata S Usami S Minami T Yamada Y Shibata J Nishikimi T Ueshima K Nakao K Increased expression of HCN channels in the ventricular myocardium contributes to enhanced arrhythmicity in mouse failing hearts. J Am Heart Assoc. 2013; 2:e000150 23709563 10.1161/JAHA.113.000150 PMC3698776

[3] DiFrancesco D The role of the funny current in pacemaker activity. Circ Res. 2010; 106:434-446 20167941 10.1161/CIRCRESAHA.109.208041

[4] Cerbai E Pino R Sartiani L Mugelli A Influence of postnatal‐development onI(f) occurrence and properties in neonatal rat ventricular myocytes. Cardiovasc Res. 1999; 42:416-423 10533577 10.1016/s0008-6363(99)00037-1

[5] Cerbai E Pino R Porciatti F Sani G Toscano M Maccherini M Giunti G Mugelli A Characterization of the hyperpolarization‐activated current, I(f), in ventricular myocytes from human failing heart. Circulation. 1997; 95:568-571 9024140 10.1161/01.cir.95.3.568

[6] Stillitano F Lonardo G Zicha S Varro A Cerbai E Mugelli A Nattel S Molecular basis of funny current (If) in normal and failing human heart. J Mol Cell Cardiol. 2008; 45:289-299 18556018 10.1016/j.yjmcc.2008.04.013

[7] Fernandez‐Velasco M Goren N Benito G Blanco‐Rivero J Bosca L Delgado C Regional distribution of hyperpolarization‐activated current (If) and hyperpolarization‐activated cyclic nucleotide‐gated channel mRNA expression in ventricular cells from control and hypertrophied rat hearts. J Physiol. 2003; 553:395-405 14514868 10.1113/jphysiol.2003.041954 PMC2343563

[8] Cerbai E Sartiani L DePaoli P Pino R Maccherini M Bizzarri F DiCiolla F Davoli G Sani G Mugelli A The properties of the pacemaker current I(F)in human ventricular myocytes are modulated by cardiac disease. J Mol Cell Cardiol. 2001; 33:441-448 11181013 10.1006/jmcc.2000.1316

[9] Hofmann F Fabritz L Stieber J Schmitt J Kirchhof P Ludwig A Herrmann S Ventricular HCN channels decrease the repolarization reserve in the hypertrophic heart. Cardiovasc Res. 2012; 95:317-326 22652004 10.1093/cvr/cvs184

[10] Luo X Lin H Pan Z Xiao J Zhang Y Lu Y Yang B Wang Z Down‐regulation of miR‐1/miR‐133 contributes to re‐expression of pacemaker channel genes HCN2 and HCN4 in hypertrophic heart. J Biol Chem. 2008; 283:20045-20052 18458081 10.1074/jbc.M801035200

[11] Kuwahara K Saito Y Takano M Arai Y Yasuno S Nakagawa Y Takahashi N Adachi Y Takemura G Horie M Miyamoto Y Morisaki T Kuratomi S Noma A Fujiwara H Yoshimasa Y Kinoshita H Kawakami R Kishimoto I Nakanishi M Usami S Saito Y Harada M Nakao K NRSF regulates the fetal cardiac gene program and maintains normal cardiac structure and function. EMBO J. 2003; 22:6310-6321 14633990 10.1093/emboj/cdg601 PMC291842

[12] Postea O Biel M Exploring HCN channels as novel drug targets. Nat Rev Drug Discov. 2011; 10:903-914 22094868 10.1038/nrd3576

[13] Fox K Ford I Steg PG Tendera M Ferrari R Ivabradine for patients with stable coronary artery disease and left‐ventricular systolic dysfunction (BEAUTIFUL): a randomised, double‐blind, placebo‐controlled trial. Lancet. 2008; 372:807-816 18757088 10.1016/S0140-6736(08)61170-8

[14] Swedberg K Komajda M Bohm M Borer JS Ford I Dubost‐Brama A Lerebours G Tavazzi L Ivabradine and outcomes in chronic heart failure (SHIFT): a randomised placebo‐controlled study. Lancet. 2010; 376:875-885 20801495 10.1016/S0140-6736(10)61259-7

[15] Tardif JC O'Meara E Komajda M Bohm M Borer JS Ford I Tavazzi L Swedberg K Effects of selective heart rate reduction with ivabradine on left ventricular remodelling and function: results from the SHIFT echocardiography substudy. Eur Heart J. 2011; 32:2507-2515 21875858 10.1093/eurheartj/ehr311 PMC3195263

[16] Cappato R Castelvecchio S Ricci C Bianco E Vitali‐Serdoz L Gnecchi‐Ruscone T Pittalis M De AL Baruscotti M Gaeta M Furlanello F Di FD Lupo PP Clinical efficacy of ivabradine in patients with inappropriate sinus tachycardia: a prospective, randomized, placebo‐controlled, double‐blind, crossover evaluation. J Am Coll Cardiol. 2012; 60:1323-1329 22981555 10.1016/j.jacc.2012.06.031

[17] Milliez P Messaoudi S Nehme J Rodriguez C Samuel JL Delcayre C Beneficial effects of delayed ivabradine treatment on cardiac anatomical and electrical remodeling in rat severe chronic heart failure. Am J Physiol Heart Circ Physiol. 2009; 296:H435-H441 19074674 10.1152/ajpheart.00591.2008

[18] Ceconi C Comini L Suffredini S Stillitano F Bouly M Cerbai E Mugelli A Ferrari R Heart rate reduction with ivabradine prevents the global phenotype of left ventricular remodeling. Am J Physiol Heart Circ Physiol. 2011; 300:H366-H373 20952661 10.1152/ajpheart.01117.2009

[19] Navaratnarajah M Ibrahim M Siedlecka U van Doom C Shah A Gandhi A Dias P Sarathchandra P Yacoub MH Terracciano CM Influence of ivabradine on reverse remodelling during mechanical unloading. Cardiovasc Res. 2013; 97:230-239 23079200 10.1093/cvr/cvs318

[20] Roubille F Tardif JC New therapeutic targets in cardiology: heart failure and arrhythmia: HCN channels. Circulation. 2013; 127:1986-1996 23671179 10.1161/CIRCULATIONAHA.112.000145

